# Acute Presentation of Extra-Pulmonary Tuberculosis in the Emergency Department: A Case Report

**DOI:** 10.7759/cureus.52142

**Published:** 2024-01-11

**Authors:** Preethy Koshy, Gajanan Chavan, Charuta Gadkari, Nigil Kuttan, Anmol K Nagpal, Rajshree Devi Seram

**Affiliations:** 1 Emergency Medicine, Jawaharlal Nehru Medical College, Datta Meghe Institute of Higher Education and Research, Wardha, IND; 2 Emergency Medicine, Jawaharlal Nehru Medical College, Datta Meghe Institiute of Higher Education and Research, Wardha, IND; 3 Emergency Medicine, awaharlal Nehru Medical College, Datta Meghe Institiute of Higher Education and Research, Wardha, IND

**Keywords:** emergency department, multi-drug resistance, metacarpal tuberculosis, pocus, pericardiocentesis, cardiac tamponade, pericardial effusion, extra-pulmonary tuberculosis

## Abstract

Pericardial effusion is a rare manifestation of tuberculosis (TB) that can present as a life-threatening emergency. It poses a diagnostic challenge, as its clinical presentation may mimic other more common causes of acute cardiac emergencies. Emergency physicians should maintain a high index of suspicion for tuberculosis, particularly in regions where the prevalence of the disease is high. This case report is about a 17-year-old girl who presented to the emergency room with dyspnea, chest discomfort, and hemodynamic instability consistent with cardiac tamponade. Urgent diagnostic procedures, including point-of-care ultrasound (POCUS) and pericardiocentesis, were crucial to the successful management of this patient.

## Introduction

Tuberculosis (TB), caused by Mycobacterium tuberculosis, has been a major public health concern for centuries. It is mainly classified into pulmonary and extra-pulmonary tuberculosis. Extra-pulmonary TB usually affects bones, the intestine, the pleura, and lymph nodes, but rarely the cardiovascular system. Cardiac tamponade is rare, even in patients with cardiac manifestations of TB [1]. Urgent drainage of the pericardial fluid is required in patients with impending tamponade to avoid clinical devastation and sudden cardiac arrest [2]. Although a diagnosis of pericardial effusion is made in the emergency department (ED), identifying patients with impending cardiac tamponade requiring immediate drainage remains challenging [3]. Here, we report a 17-year-old girl with multidrug-resistant (MDR) TB of metacarpal bone who presented to the emergency department with pericardial effusion in cardiac tamponade.

## Case presentation

A 17-year-old girl was brought to the emergency room of our hospital with the main complaints of chest discomfort, progressive breathlessness, and multiple episodes of vomiting of two days duration. Her history was significant for MDR TB of the right second metacarpal bone, diagnosed eight months ago, and she was on antituberculosis treatment (ATT).

**Figure 1:**
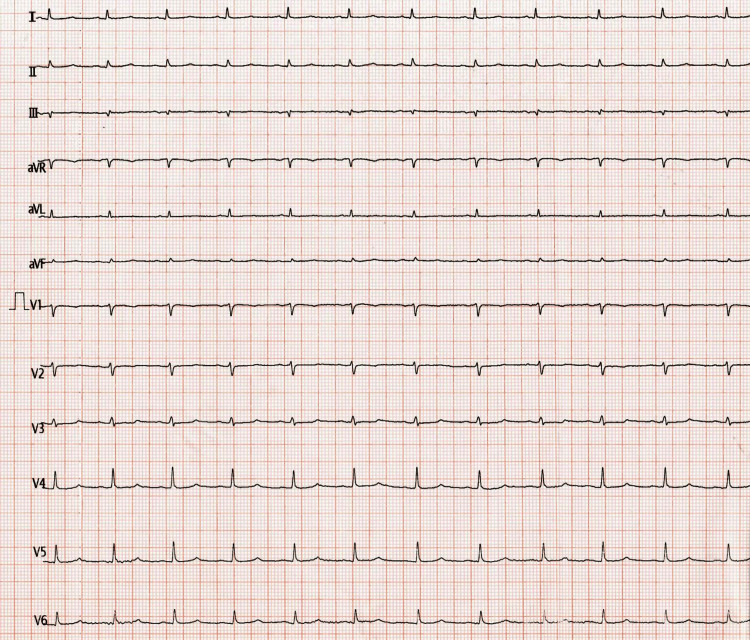
ECG showing low voltage complexes

On primary assessment, the patient was confused and irritable. She was tachypneic, with a respiratory rate of 30/min and an oxygen saturation of 96% on room air. She had cold clammy extremities with a low volume pulse (rate of 94/min), a blood pressure of 80/50 mm of Hg, and prolonged capillary refill time. Her random blood sugar was 22 mg/dl, which was corrected with intravenous glucose. Examination of the cardiovascular system revealed soft heart sounds with raised jugular venous pressure (JVP) and distended neck veins. Bilateral basal crepitations were present on auscultation of the chest. An electrocardiogram (ECG) revealed low-voltage complexes, as shown in Figure 1.

**Figure 2:**
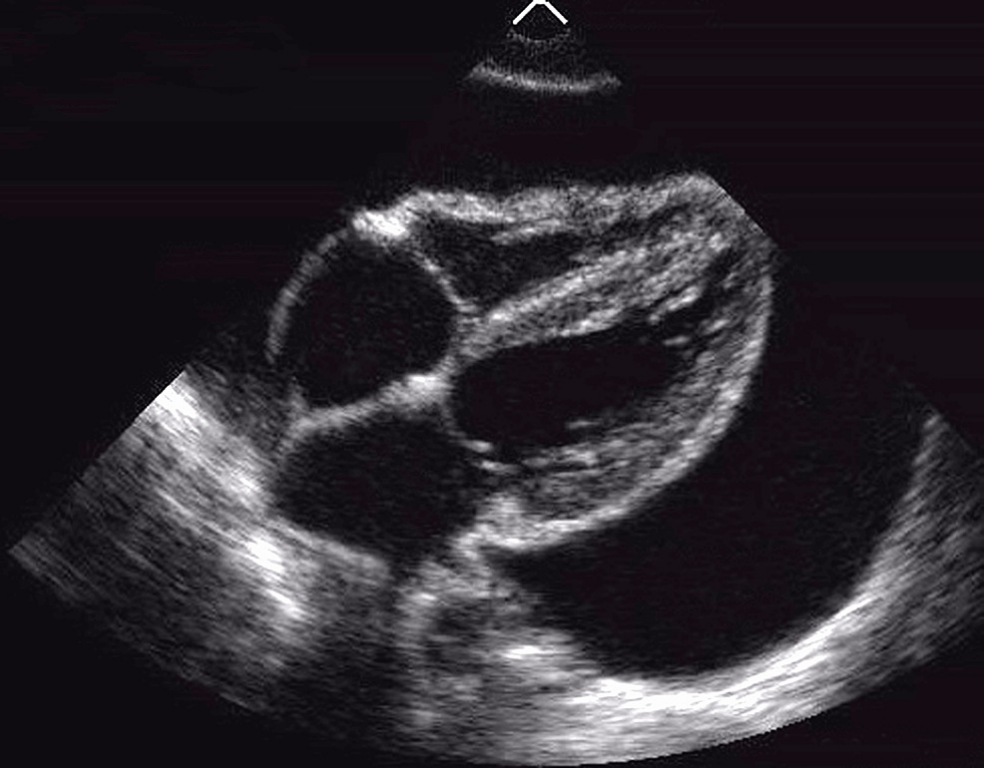
Point of care ultrasound showing pericardial effusion

Suspecting pericardial effusion, a point-of-care ultrasound (POCUS) was done, which confirmed the diagnosis as shown in Figure 2.

**Figure 3:**
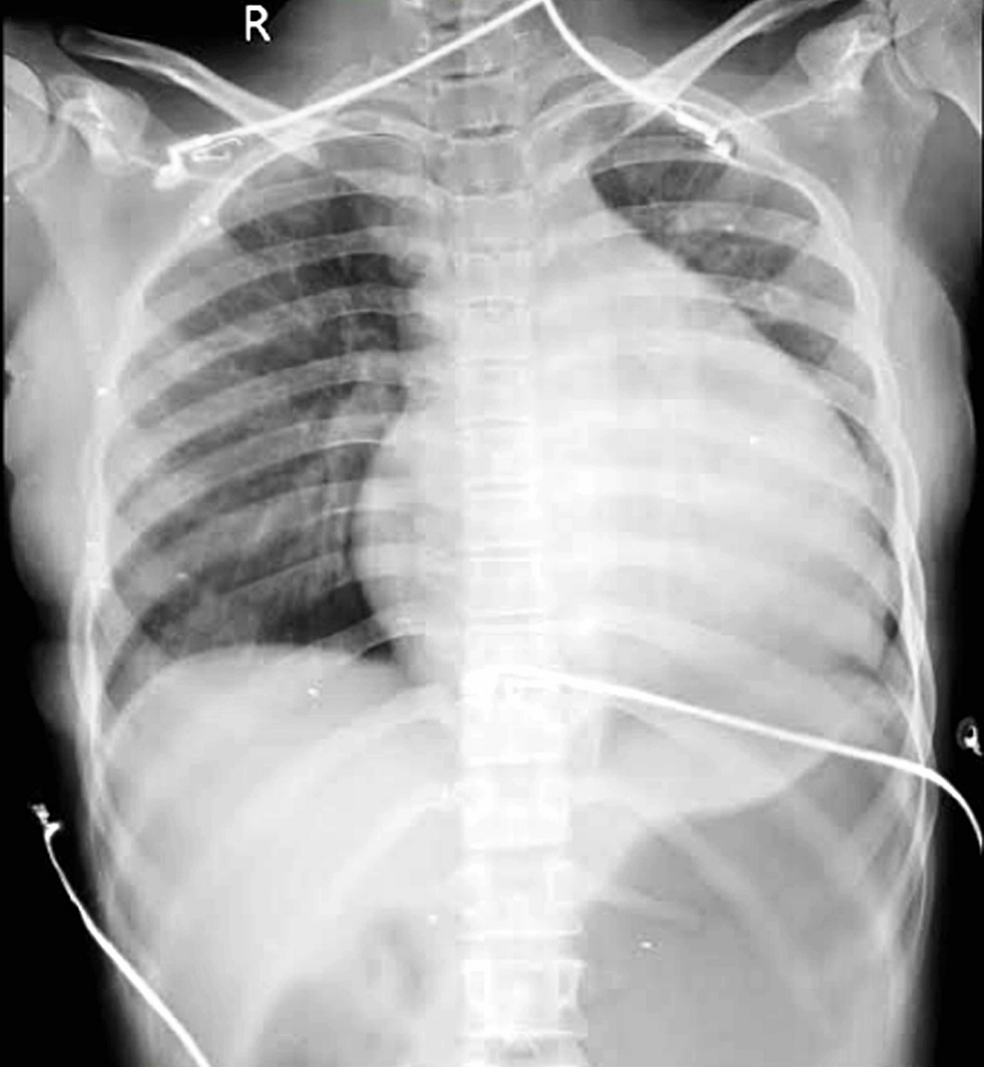
Chest X-ray of patient showing cardiomegaly

A bedside chest X-ray done showed cardiomegaly, which added to the diagnosis, as evident in Figure 3.

Her arterial blood gas analysis showed severe metabolic acidosis, as shown in Table 1.

**Table 1:** Arterial blood gas analysis of patient showing severe metabolic acidosis pH: potential of hydrogen, pCO_2_: partial pressure of carbon dioxide, pO_2_: partial pressure of oxygen

Parameters	Patient’s value	Normal range
pH	6.7	7.35- 7.45
pCO_2_	12.4 mm Hg	35-45 mm Hg
pO_2_	106 mm Hg	80-100 mm Hg
Base	3.3 mmol/L	22-26 mmol/L

**Figure 4:**
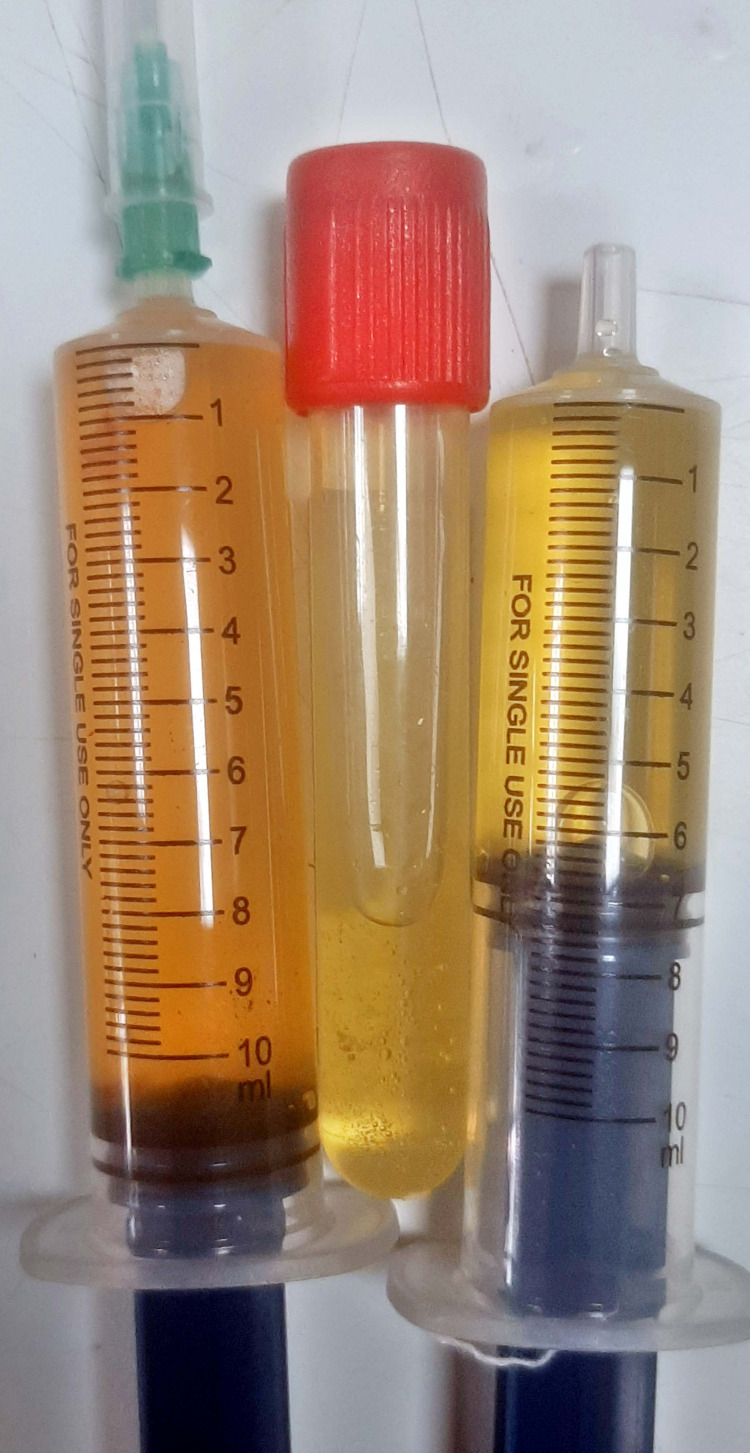
Serous pericardial fluid aspirated from the patient

Bedside ultrasound-guided pericardiocentesis was done via a sub-xiphoid approach, draining 100 ml of serous fluid as shown in Figure 4.

She was then managed with intravenous inotropes, sodium bicarbonate, oxygen supplementation, and other supportive measures. Her sensorium improved gradually and she maintained a blood pressure of 110/70 mm Hg. Inotropic support was tapered and she was shifted to the intensive care unit, where a 2D echo showed mild to moderate pericardial effusion, 13 mm to the right and 10 mm to the left, not suggestive of tamponade. The pigtail was inserted and around 380 ml of fluid was further removed. Pericardial fluid analysis showed no organisms or pus cells; however, adenosine deaminase (ADA) was elevated (46 µ/l). A repeat 2D echo done after one week showed mild residual pericardial fluid. She became symptomatically better; inotropes were tapered and stopped and she was discharged home.

## Discussion

Pericardial effusion may have many causes, including malignancy, infections, mediastinal radiotherapy, and idiopathic causes [4]. Among all cases of extra-pulmonary TB, tuberculous pericardial effusion has an estimated incidence of up to 2% [5]. Hypotension, elevated JVP, and muffled heart sounds (Becks triad) seen in this patient were highly suspicious of pericardial effusion. Low-voltage complexes seen in the ECG, POCUS, and bedside chest X-ray confirmed the diagnosis. Performing ultrasound-guided pericardiocentesis in the emergency department was a lifesaving intervention in this case.

Determining the cause of tamponade proved to be challenging. Some studies show that the cause of pericardial diseases remains undetermined in 15-20% of cases [6]. This girl has had MDR TB of the right second metacarpal bone for eight months and has been on treatment with ATT for the same since then. So, the cause of pericardial effusion was attributed to TB. The diagnosis of tubercular pericarditis involves the detection of tubercle bacilli in pericardial fluid or the presence of TB elsewhere in the patient with otherwise unexplained pericarditis, a lymphocyte-predominant pericardial exudate with a raised ADA level, and/or appropriate response to ATT [7]. Pericardial fluid analysis, in this case, showed lymphocytic predominance with elevated ADA and no organisms. She became symptomatically better with timely pericardiocentesis and ATT and was discharged home.

## Conclusions

Pericardiocentesis should be considered an urgent procedure in the ED, as it can be life-saving when cardiac tamponade is suspected or diagnosed. High suspicion for the condition, clinical signs and symptoms, along with imaging studies like echocardiography, help to confirm the diagnosis of cardiac tamponade in the ED. Thus, POCUS has revolutionized the way ED physicians assess and manage patients, as it provides real-time ultrasound images at the bedside. TB of the metacarpal bone presenting with pericardial effusion is not much seen in the literature. Tuberculosis, being a persistent global health challenge, requires continued research and investment to further reduce its impact on individuals and communities.

## References

[1] Shakya S, Jha SC (2018). Cardiac manifestations of tuberculosis in a tertiary care center of Nepal. Nepal Heart J.

[2] Spodick DH (2003). Acute cardiac tamponade. N Engl J Med.

[3] Argulian E, Messerli F (2013). Misconceptions and facts about pericardial effusion and tamponade. Am J Med.

[4] Hussein AM, Korkmaz UT, Yapıcı K, Ali AA, Kizilay M (2021). Successfully managed case of cardiac tamponade due to tuberculous pericardial effusion: a case study. Iran Heart J.

[5] Trautner BW, Darouiche RO (2001). Tuberculous pericarditis: optimal diagnosis and management. Clin Infect Dis.

[6] Noubiap JJ, Agbor VN, Ndoadoumgue AL, Nkeck JR, Kamguia A, Nyaga UF, Ntsekhe M (2019). Epidemiology of pericardial diseases in Africa: a systematic scoping review. Heart.

[7] Mayosi BM, Burgess LJ, Doubell AF (2005). Tuberculous pericarditis. Circulation.

